# Supplementary material for: A comparison of sequencing platforms and bioinformatics pipelines for compositional analysis of the gut microbiome
Source: BMC Microbiol. 2017 Sep 13;17:194. doi: 10.1186/s12866-017-1101-8 (PMC5598039; doi:10.1186/s12866-017-1101-8)
Supplement: Supplementary file 1 — Table S1. Phylogenetic diversity (PD) and species richness (S) values by platform, experimental group, and bioinformatics pipeline. Table S2. Most abundant phyla identified by platform, experimental group, and bioinformatics pipeline. (DOCX 159 kb) [file 12866_2017_1101_MOESM1_ESM.docx]

**A comparison of sequencing platforms and bioinformatics pipelines for compositional analysis of the gut microbiome**

Imane Allali^1^*, Jason W. Arnold^1^ Jeffrey Roach^2^, Maria Belen Cadenas^1^, Natasha Butz^1^, Hosni M. Hassan^3^, Matthew Koci^3^, Anne Ballou^3^, Mary Mendoza^3^, Rizwana Ali^3^, and M. Andrea Azcarate-Peril^1#^

^1^Department of Medicine, Division of Gastroenterology and Hepatology, and Microbiome Core Facility, Center for Gastrointestinal Biology and Disease, School of Medicine, University of North Carolina, Chapel Hill, NC

^2^Research Computing, University of North Carolina, Chapel Hill, NC

^3^Department of Poultry Science, North Carolina State University, Raleigh, NC

*Current address: Laboratory of Biochemistry & Immunology, Faculty of Sciences, Mohammed V University, Rabat, Morocco

^#^To whom correspondence should be addressed:

Department of Medicine

332 Isaac Taylor Hall - Campus Box 7555

School of Medicine

University of North Carolina

Chapel Hill, NC 27599-7545

Email: [azcarate@med.unc.edu](mailto:azcarate@med.unc.edu)

Phone: 919 966 9838

Imane Allali [allali.imane@gmail.com](mailto:allali.imane@gmail.com)

Jason William Arnold [jason_arnold@med.unc.edu](mailto:jason_arnold@med.unc.edu)

Jeffrey Roach [jeff_roach@unc.edu](mailto:jeff_roach@unc.edu)

María Belén Cadenas [mbcade@yahoo.com](mailto:mbcade@yahoo.com)

Butz, Natasha [natasha_butz@med.unc.edu](mailto:natasha_butz@med.unc.edu)

Hosni Hassan [hmhassan@ncsu.edu](mailto:hmhassan@ncsu.edu)

Matthew Koci [mdkoci@ncsu.edu](mailto:mdkoci@ncsu.edu)

Anne Ballou [alballou@ncsu.edu](mailto:alballou@ncsu.edu)

Rizwana Ali [riz@ncsu.edu](mailto:riz@ncsu.edu)

Mary Mendoza [mamendoz@ncsu.edu](mailto:mamendoz@ncsu.edu)

M. Andrea Azcarate-Peril [azcarate@med.unc.edu](mailto:azcarate@med.unc.edu)

**Abstract**

**Background**: Advancements in Next Generation Sequencing (NGS) technologies regarding throughput, read length and accuracy had a major impact on microbiome research by significantly improving 16S rRNA amplicon sequencing. As rapid improvements in sequencing platforms and new data analysis pipelines are introduced, it is essential to evaluate their capabilities in specific applications. The aim of this study was to assess whether the same project-specific biological conclusions regarding microbiome composition could be reached using different sequencing platforms and bioinformatics pipelines.

**Results**: Chicken cecum microbiome was analyzed by 16S rRNA amplicon sequencing using Illumina MiSeq, Ion Torrent PGM, and Roche 454 GS FLX Titanium platforms, with standard and modified protocols for library preparation. We labeled the bioinformatics pipelines included in our analysis QIIME1 and QIIME2 (*de novo* OTU picking [not to be confused with QIIME version 2 commonly referred to as QIIME2]), QIIME3 and QIIME4 (open reference OTU picking), UPARSE1 and UPARSE2 (each pair differs only in the use of chimera depletion methods), and DADA2 (for Illumina data only). GS FLX+ yielded the longest reads and highest quality scores, while MiSeq generated the largest number of reads after quality filtering. Declines in quality scores were observed starting at bases 150-199 for GS FLX+ and bases 90-99 for MiSeq. Scores were stable for PGM-generated data. Overall microbiome compositional profiles were comparable between platforms; however, average relative abundance of specific taxa varied depending on sequencing platform, library preparation method, and bioinformatics analysis. Specifically, QIIME with *de novo* OTU picking yielded the highest number of unique species and alpha diversity was reduced with UPARSE and DADA2 compared to QIIME.

**Conclusions:** The three platforms compared in this study were capable of discriminating samples by treatment, despite differences in diversity and abundance, leading to similar biological conclusions. Our results demonstrate that while there were differences in depth of coverage and phylogenetic diversity, all workflows revealed comparable treatment effects on microbial diversity. To increase reproducibility and reliability and to retain consistency between similar studies, it is important to consider the impact on data quality and relative abundance of taxa when selecting NGS platforms and analysis tools for microbiome studies.

**Keywords**

16S rRNA amplicon sequencing - microbiome analysis - microbiome - microbiome composition - next generation sequencing platforms – bioinformatics pipeline – NGS bias

**Background**

Sequencing of 16S rRNA amplicons is now a well-established and robust method used in compositional studies of the gut microbiome of humans, animals, and insects. These studies have generated a wealth of information regarding the impact of diet [1-4], disease [5-7], antibiotics [8], probiotics [9-11], prebiotics [12, 13] and environmental exposures [14] on the microbiota, while simultaneously permitting the identification of new bacteria [15]. However, the excitement of applying these emerging technologies to new and important research questions has relegated important technical considerations affecting comparisons between research done by different laboratories following different protocols. Given the importance of NGS technologies on microbiome research, comparative studies focusing on different platforms are essential to confirm or refute published data. Early microbiome studies focused on quality control aimed to reduce sequencing error by bioinformatically removing chimeras and other sequencing artifacts from 16S rRNA amplicon sequences generated by pyrosequencing [16]. Later, studies have compared data output from different sequencing platforms applied to genome [17-19] and 16S rRNA amplicon sequencing [20, 21]. While chimera removing bioinformatics tools can reduce or eliminate chimeric sequences in a dataset, it is challenging to eliminate the bias introduced by primer design [22], library preparation [23], DNA isolation methods [24], and PCR amplification artifacts, each of which introduce unique biases that can result in over or underrepresentation of individual microbes within complex communities [25]. These biases are unavoidable and rarely impact the overall merit of a study.

Collective concerns regarding methodological biases and the ability to compare studies from different research groups originated in a collaborative effort designed to comprehensively evaluate methods employed in the study of the human microbiome, the Microbiome Quality Control project (MBQC). Inspired by earlier projects like the Microarray Quality Control project (MAQC), the MBQC focused on the analyses of the impacts of sample collection, DNA extraction, sequencing protocol, and bioinformatics data analysis pipelines on amplicon profiling of the human fecal microbiome. Although the coalition is still in early stages, the MBQC has identified the DNA isolation method (and the lab performing the DNA isolation), as well as 16S rRNA amplification primers used, as major sources of variation, while sequencing depth and sample storage had small but detectable effects on the generated data [26].

A number of recent studies have attempted to identify errors or bias generated by the intrinsic characteristics of sequencing platforms [18, 27, 28]. Performance comparisons between sequencing platforms and bioinformatics pipelines indicate that Roche GS FLX+, Illumina MiSeq, and Ion Torrent PGM are capable of generating high quality, comparable data [22, 29-31]. In one study, the performance of Ion Torrent PGM, Pacific Biosciences RS and Illumina MiSeq platforms was compared on genome sequencing of 4 bacterial strains with different GC contents [17]. Although all three platforms provided sufficient depth and resolution, there were biases present in each platform. PGM yielded deep coverage for GC-rich sequences but was biased for AT-rich sequences coverage. PacBio and MiSeq demonstrated equivalent coverage of GC- and AT-rich sequences [17], but had varying error rates prior to assembly [32]. A different study compared the performances of Illumina GA II and Roche GS FLX+ using the same DNA samples obtained from a complex freshwater planktonic community. The platforms showed comparable total diversity; however, more homopolymer errors were identified with the Roche GS FLX+ platform compared to GA II which generated longer and more accurate contigs [18]. Finally, a comparison of the healthy skin microbiome using the Illumina MiSeq and Roche GS FLX+ platforms showed that sequencing data from the V3-V4 16S rRNA hypervariable region were concordant between replicates, and between platforms indicating that the method and platforms were comparable for determining skin microbiota [33].

Further comparative studies between Ion Torrent PGM, Illumina MiSeq, Illumina HiSeq, and Roche GS FLX+ confirmed that the later generated the longest reads among these platforms (up to 600 bp) but had a relatively high error rate with poly-bases of more than 6 base pairs [27]. Additionally, sequencing runs on Roche GS FLX+ had a higher cost and lower throughput than the Illumina platform. Conversely, the Illumina platform had the fastest run time and highest throughput, up to 13.5 Gb on the MiSeq PE300, but relatively high frequency of substitution errors and shorter reads compared to GS FLX+ [34]. Run time and homopolymer error rates for the Ion Torrent PGM platform was substantially lower compared to the Roche GS FLX+ platform but yielded a lower throughput, shorter reads and lower quality scores [35].

Together, the previously discussed data indicate that technical protocols and sequencing platforms have a variable impact on output. In this study, we report a comparison of the three most widely utilized sequencing platforms: Illumina MiSeq, Ion Torrent PGM, and Roche 454 GS FLX+, using the most current library preparation protocols and sequencing kits available for 16S rRNA amplicon sequencing. Although Roche discontinued support for the 454 GS FLX+ sequencing platform in 2016, the platform still has relevance to studies that have used this technology in the past or any ongoing studies that may utilize a sequencing provider whom is still running the 454 GS FLX+ sequencer. More importantly, this study couples the platform comparison with a systematic assessment of bioinformatics pipelines commonly used for amplicon data analysis: Quantitative Insights Into Microbial Ecology (QIIME)[36] and UPARSE[37] with or without chimera detection methods, based either on *de novo* OTU picking or open reference OTU picking. Although a relatively recently developed tool that has not been extensively tested for accuracy and efficacy, we have also included DADA2 [38] and a comparator method in our analysis in order to assess the effectiveness of bioinformatic analysis at a finer level than the traditional 97% similarity threshold.

Fourteen cecum samples randomly selected from an ongoing study aiming to investigate the impact of vaccination against *Salmonella* and prebiotic supplementation on the chicken gut microbiome and immune responses were used in this study. We have previously shown that prebiotics have a modulatory effect on the gut microbiome [13, 39], not by dramatically altering its composition but by impacting very specific bacterial groups. Hence, we chose to include in our comparison of bioinformatics pipelines methods without chimera removal in order to determine if specific groups known to be altered by the prebiotic (*Bifidobacterium, Lactobacillus*) were impacted by this additional step. Clearly, the utility of chimera removal has been widely demonstrated in the analysis of 16S rRNA amplicon sequencing data [40-42].

The aim of the present study was to explore influences of sequencing platforms and bioinformatics pipelines on diversity and relative abundance of bacterial taxa in 16S rRNA amplicon data. Additionally, each platform and analysis pipeline was compared for their abilities to discriminate between samples from various treatment groups in order to validate their functionality in microbiome studies.

**Methods**

**Samples and DNA isolation**

The same physical samples were used for all sequencing experiments across platforms and bioinformatics pipelines. Total genomic DNA was extracted using E.Z.N.A. Stool DNA Kit (Omega Bio-Tek, Norcross, GA) according to manufacturer’s instructions with minor modifications. Briefly**,** 200 mg of intestinal content were added to a tube containing 540 µl of SLB buffer and 200 mg of glass beads. Samples were homogenized using a TissueLyser (Qiagen, Germantown, MD) for 5 minutes at 30 Hz in 1 minute intervals between bead beating and ice incubation cycles. DS buffer and proteinase K were added according to the manufacturer’s instruction. The mix was incubated at 70°C for 10 minutes, followed by another incubation at 95°C for 5 minutes. Quality of the isolated DNA was assessed by agarose gel electrophoresis and purity verified using 260/280 and 260/230 ratios measured by NanoDrop 1000 instrument (Thermo Fisher Scientific, Waltham, MA). DNA concentration was quantified using Quant-iT™ PicoGreen dsDNA Reagent (Molecular Probes, Thermo Fisher Scientific division, Eugene, OR).

**454 Genome Sequencer FLX+ 16S rRNA amplicon sequencing**

Protocols for preparation of libraries for sequencing are represented in Figure 1. Initial amplification of the hypervariable V1-V2 region of the bacterial 16S rRNA was performed on total DNA from collected samples as previously described [1, 13, 43]. Reaction master mixes contained the Qiagen Hotstar Hi-Fidelity Polymerase kit reagents (Qiagen, Valencia CA) with a forward primer composed of the Roche Titanium Fusion Primer A (sequencing primers used in this study are listed in Table 1) a 10bp Multiplex Identifier (MID) sequence (Roche, Indianapolis, IN), unique to each of the samples, and the universal bacterial primer 8F [44]. The reverse primer was composed of the Roche Titanium Primer B, the identical 10bp MID sequence, as the forward primer, and the reverse bacterial primer 338R [45]. Negative controls, not containing template, were amplified for all barcode-primer sets. Each sample was gel purified individually using the E-Gel Electrophoresis System (Life Technologies, Thermo Fisher Scientific division, Grand Island, NY) and standardized prior to pooling. The 16S rRNA amplicons were sequenced on a 454 Genome Sequencer FLX+ system instrument (Roche, Indianapolis, IN) at the Microbiome Core Facility, (University of North Carolina, Chapel Hill, NC) using the GS FLX Titanium XLR70 sequencing reagents and corresponding protocol. Initial data analysis, base pair calling and trimming of each sequence to yield high quality reads, were performed by Research Computing at the University of North Carolina at Chapel Hill.

**Illumina MiSeq 16S rRNA amplicon sequencing**

DNA was amplified using primers targeting the V1-V2 region of the bacterial 16S rRNA gene [15, 44] and overhang adapter sequences appended to the primer pair for compatibility with Illumina index and sequencing adapters. The complete sequences of the primers are listed in Table 1. Master mixes contained 12.5 ng of total DNA and 2x KAPA HiFi HotStart ReadyMix (KAPA Biosystems, **Wilmington, MA**). Negative controls, not containing template, were amplified for all barcode-primer sets. Each 16S amplicon was purified using AMPure XP reagent (Beckman Coulter, Brea, CA). In the next step each sample was amplified using a limited cycle PCR program, adding Illumina sequencing adapters and dual- index barcodes (index 1(i7) and index 2(i5)) **(Illumina, San Diego, CA)** to the amplicon target. The final libraries were again purified using AMPure XP reagent (Beckman Coulter), quantified and normalized prior to pooling. The DNA library pool was then denatured with NaOH, diluted with hybridization buffer and heat denatured before loading on the MiSeq reagent cartridge **(Illumina) and on the MiSeq instrument (Illumina). Automated cluster generation and paired-end sequencing with dual reads were performed** according to the manufacturer’s instructions.

**Ion Torrent PGM 16S rRNA amplicon sequencing**

For amplicon library preparation the V1-V2 hypervariable region of the 16S rRNA gene was amplified from total bacterial DNA using the forward primer composed of Ion Torrent adapter A, a 10bp IonXpress ™ barcode (Life Technologies, Thermo Fisher Scientific division, Grand Island, NY), unique to each sample and the universal bacterial primer 8F. The reverse primer consisted of Ion Torrent trP1 adapter followed by reverse bacterial primer 338R. PCR reactions contained 50 ng of DNA template, 2.5 units of HotStar Hi-fidelity DNA polymerase (Qiagen, Valencia, CA), 1x HotStar Hi-Fidelity PCR buffer containing dNTPs, and 0.6 µM of each primer. Negative controls, not containing template, were amplified for all barcode-primer sets. The PCR products were gel purified individually using the E-Gel Electrophoresis System. DNA concentrations were quantified using Quant-iT™ PicoGreen® dsDNA Reagent (Molecular Probes, Thermo Fisher Scientific division, Eugene, OR) and mixed at equimolar concentrations. Template-Positive Ion OneTouch™ 200 Ion Sphere™ Particles were prepared from library pool using the Ion OneTouch™ 2 system (Life Technologies, Thermo Fisher Scientific division, Grand Island, NY). The prepared templates were sequenced on the Ion Torrent PGM instrument (Life Technologies) in the Microbiome Core Facility using the Ion PGM 400 sequencing reagents. All kits were used according to the manufacturer’s instructions. Initial data analysis, base pair calling and trimming of each sequence was performed on Ion Torrent browser to yield high quality reads.

**Modifications to the library preparation methodologies**

**The library preparation protocol for the three evaluated sequencing platforms is depicted in Figure1. The Illumina amplification protocol for 16S rRNA amplicon generation has 25 cycles at 55°C annealing temperature, while the protocol used in this study for barcoding and library preparation for 454 and Ion Torrent has 10 more cycles and 5 degrees less in the annealing temperature step of the PCR reaction. Therefore, we decided to evaluate modifications of the PGM library preparation protocol to determine if these differences had an impact on diversity and taxa composition of samples. Consequently, for the second PGM run (PGM2) we maintained the same annealing temperature (50ºC) but reduced the number of cycles to 25 and for the third PGM run (PGM3) we maintained the number of cycles (35 cycles) but increased the annealing temperature to 55ºC.**

**Bioinformatics analysis**

**Roche 454 sequencing results were initially processed using GS Data Analysis Software package [46]. The three Ion Torrent sequencing runs (PGM1, PGM2 and PGM3) were initially processed using the onboard data analysis software of the Ion PGM [47]. The two Illumina sequencing runs (MiSeq1 and MiSeq2) were converted to multiplexed fastq format using CASAVA 1.8.2 [48]. Paired-end reads from the Illumina platform were joined using the QIIME 1.8.0 [36] invocation of fastq-join [49].** Bioinformatic analysis of bacterial 16S rRNA amplicon data was conducted using the QIIME software pipeline [36] and as described [43]. The combined six raw sequencing data plus metadata describing the samples were de-multiplexed and filtered for quality control. Sequences were aligned and clustered into operational taxonomic units (OTU) based on the *de novo* OTU picking algorithm using the QIIME implementation of UCLUST [50]. After OTU picking step [50], chimeras and singletons were removed using ChimeraSlayer [41, 51]. After taxonomic assignation of OTUs, sequences were aligned and phylogenetic trees were built with FastTree 2.1.3 [52]. OTUs with 97% similarity level were selected for taxonomical assignment and employed for diversity (Shannon index) and richness analysis. Beta diversity estimates were calculated within QIIME using weighted and unweighted Unifrac distances [53] between samples at a sub-sampling depth of 1,000 sequences per sample. From these estimates, jackknifed principal coordinates were computed to compress dimensionality into two- and three*-*dimensional principal coordinate analysis plots. QIIME was also used to calculate alpha diversity with a sub-sampling depth of 1000 using observed species*,* Shannon and phylogenetic diversity (PD) metrics. To evaluate the similarities between bacterial communities a principal coordinate analysis (PCoA) using Unweighted and Weighted Fast Unifrac [53, 54] was performed to compare samples based on their treatment.

In addition to the primary bioinformatics pipeline, additional pipelines were applied. The first pipeline was based on the *de novo* OTU picking algorithm as implemented in QIIME 1.8.0 [50] as the primary bioinformatics pipeline however in this pipeline chimeras were not removed. The second and third pipelines were based on open reference OTU picking as implemented in QIIME 1.8.0 [50] and differ only in the use of ChimeraSlayer [41, 51]. The fourth and fifth pipeline was based on UPARSE[37] and differ only in the use of chimera detection. The different bioinformatics pipelines were named as follows; QIIME1 refers to the pipeline based on the *de novo* OTU picking without chimera checking, QIIME2 (not to be confused with QIIME version 2 commonly referred to as QIIME2), the *de novo* OTU picking pipeline with chimera detection, QIIME3 refers to the open reference OTU picking pipeline without chimera checking, and QIIME4 the open reference OTU picking pipeline with chimera detection. In addition, two pipelines based on UPARSE were used: UPARSE1 with chimera detection and UPARSE2 without chimera checking (Figure 2).

Restricting consideration to the two Illumina MiSeq platform results, two pipelines intended to assess microbial communities at a finer resolution than the traditional 97% similarity threshold were considered. The software package DADA2 [38] was used to produce a sequence table to be compared to an OTU table produced from QIIME *de novo* OTU picking with a similarity threshold of 99% from which chimeric sequences and singleton OTUs were filtered. Assignment of taxonomy was performed on each table using both the DADA2 taxonomy classification method and the QIIME taxonomy classification method. Beta diversity and procrustes analysis were applied to the results of both approaches for comparison. The non-phylogenetic Bray-Curtis metric was used for calculation of beta diversity.

PhyloToAST [55] analysis was performed on data and visualized using the Interactive Tree of Life (iTol) v3 [56].

**Statistical analyses**

Analysis of variance (ANOVA) was used to identify significant differences in phylogenetic diversity (PD) and species richness (S) indexes. Analysis of Similarities (ANOSIM) and Permutational Multivariate Analysis of Variance (PERMANOVA) analyses were applied to Unweighted Unifrac similarity matrices to compute similarities between groups. The Kruskal Wallis test for multiple pairwise comparisons was performed to evaluate significant differences in relative abundances of bacterial taxa using JMP genomics (SAS, JMP Genomics 10.0). In all analyses alpha was set at 0.05.

**Results**

Samples from this study are from a *Salmonella* challenge study conducted in poultry. The parent study aimed to evaluate the impact of an attenuated *Salmonella* strain on the gut microbiota of poultry, and to determine its effect on the gut microbiome and efficacy on *Salmonella* infection clearance after challenge. A secondary objective was to determine how prebiotics modify the structure of the gut microbiome, and the impact of this modification on *Salmonella* infection clearance (Azcarate-Peril et al., in preparation). In the current study, analysis of microbiome composition was conducted on 14 chicken cecum samples randomly selected from 3 different groups: 1) control group, 2) *Salmonella*-vaccinated group, and 3) prebiotics-fed group. Each of the groups was assigned also to two subgroups according to a *Salmonella* challenge. Table 2 lists the characteristics of each sample. In order to compare platforms performance and bioinformatics analysis pipelines, total DNA from samples was amplified with primers targeting the V1-V2 hypervariable region of the 16S rRNA gene and sequenced using the GS FLX+ (Roche), Ion Torrent PGM or Illumina MiSeq platforms according to the scheme shown in Figure 1. A summary of expected sequencing output, reads length, and quality scores from the three platforms is presented in Table 3. The GS FLX+ run produced the lowest number of raw reads (1/8 of a plate) compared to PGM (314X chip) and MiSeq (one lane); however, quality scores were higher in comparison to both PGM and MiSeq platforms (Table 4). Quality scores were more stable over the read length for the PGM instrument while we observed a decline starting at bases 150-199 for the GS FLX+ run and at bases 90-99 for the MiSeq run. After filtering (length > 200 bp, quality scores > 25), 96% of the GS FLX+ reads, 91.8% of the MiSeq, but only 14.9% of the PGM platform reads were suitable for further analysis. However, the filtered number of reads for this platform and sequencing chip was within the manufacturer’s indicated output (≈ 80,000 - 100,000 reads/314X chip).

**UPARSE reported a lower phylogenetic diversity than both *de novo* QIIME and open reference QIIME pipelines regardless of the sequencing platform**

The bioinformatics analysis pipelines used in our study to determine the taxonomic composition and bacterial diversity of 14 chicken cecum samples using three different platforms are depicted in Figure 2. Rarefaction analyses at an even sampling depth of 1,000 reads/sample were conducted to determine Phylogenetic Diversity (PD) and Species Richness (S) of samples (Figures 3A and 3B, Table S1). PD values were lower for all platforms when UPARSE pipelines were applied to data compared to both *de novo* QIIME pipelines and open reference QIIME pipelines (Figure 3A). A similar observation in the case of de novo OTU picking was previously reported by Pylro et al. [57]. In contrast, S values were comparable between all bioinformatics pipelines (Figure 3B). Statistically significant differences in pairwise comparisons between platforms and all bioinformatics pipelines are summarized in Figure 3, right panels. In general terms, diversity values for GS FLX+ compared to the MiSeq runs were significantly different, while diversity values for the PGM1 platform (the PGM standard method) were not significantly different to data generated using the MiSeq platform. The non-standard PGM2 and PGM3 generated diversity values were significantly different to the Illumina, GS FLX+ and standard PGM runs. Interestingly, significant differences were observed in the number of species detected between the Miseq1 and Miseq2 runs, which were intended to test reproducibility, when data was applied through QIIME1, QIIME2, and UPARSE2 pipelines. We did not observe overall statistical significant differences in phylogenetic diversity or number of species due to treatment, time, or chicken within any of the platforms.

**Overall microbiome composition of samples**

Analysis of Similarities (ANOSIM) and Permutational Multivariate Analysis of Variance (PERMANOVA) analyses were performed on Unweighted Unifrac data to evaluate differences between the 6 runs and bioinformatics pipelines. We did not observe clustering by sequencing platform (Figure S1) but instead samples grouped according to their corresponding experimental treatment group regardless of the sequencing platform and bioinformatics pipelines (Figure 4A). Principal Coordinates Analysis (PCoA) of samples also showed two sub clusters in the prebiotics group, which are most probably associated with different time points in the course of this experiment. Similar results were observed in the Principal Coordinates Analysis (PCoA) based on Weighted Unifrac matrices (not shown).

Procrustes analysis allowed us to determine whether the same conclusions could be made from different β-diversity analyses regardless of which platform was used to compare the samples. Although Procrustes analysis of data generated in the three platforms (GS FLX+, MiSeq1 and PGM1) analyzed using the QIIME2 pipeline (*de novo* OTU picking plus chimera depletion) showed substantial differences (Monte Carlo p < 0.01 and high M^2^ values) (Figure 4B), these differences were not as large as the differences observed between treatments groups. This analysis suggests that in spite of differences in the β-diversity, all platforms were capable of discriminating between treatments.

**Microbiome composition was impacted by platform and bioinformatics pipeline**

Operational Taxonomic Units (OTUs) were identified at a 97% similarity cut-off in the QIIME and UPARSE pipelines. The number of sequences assigned to OTUs for GS FLX+ was similar in all bioinformatics pipelines (99%). *De novo* (QIIME1 and QIIME2) versus open-reference OTU picking impacted the number of assigned sequences for Illumina MiSeq runs but not for GS FLX+ runs. QIIME1 and QIIME2, both using UCLUST for *de novo* OTU picking, and UPARSE1 and UPARSE2, both using USEARCH for open reference OTU picking, resulted in a higher number of unassigned reads from the Illumina MiSeq generated data (ranging from 3.6 to 4.1%). Marginal differences were observed in the OTU assignment between the three PGM runs in all bioinformatics pipelines. PGM3 (same annealing temperature, 50°C, and a reduced number of cycles from 35 to 25) showed a Firmicutes abundance of almost 99%, while PGM1 and PGM2 ranged from 96.8% to 98.7%. However, this difference was exaggerated when comparing Vaccinated and Vaccinated-Challenged samples, with Firmicutes dropping as low as 72.8% for PGM3 vs 90.3% for PGM2. (Table S2).

Firmicutes and Bacteroidetes were the most represented phyla in QIIME and UPARSE pipelines. GS FLX+ generated data showed the highest abundance of Firmicutes with an average of 96.2% followed by the MiSeq platform with an average of 93.2%, and PGM with 91.8%. Bacteroidetes were detected at 3% by GS FLX+, 2.3% by the MiSeq platform, and 6.4% by PGM. Proteobacteria and Tenericutes were represented in low abundance in all platforms and pipelines; however, they were significantly (Kruskal-Wallis *P* ≤ 0.01) over represented in the MiSeq generated data compared to PGM and GS FLX+.

Distinct differences were observed between platforms and pipelines in the relative abundance of specific genera (Figure 5). Overall, the QIIME pipelines generated a different bacterial profile compared to the UPARSE pipeline with a clear impact of the chimera depletion on relative abundance of bacterial taxa. GS FLX+ resulted in an over representation of *Eubacterium cylindroides* compared to the other platforms and protocols. Modification of PCR protocols in the PGM runs also had an impact on this taxa as well as on detection of *Butyricicoccus pullicaecorum,* a butyrate producer thought to exert anti-inflammatory effects [58] and *Oscillospira*, a genus highly represented in the data generated by the PGM platform and analyzed by the QIIME pipeline with no chimera depletion (QIIME1). The open reference OTU picking pipelines (QIIME3 and QIIME4) generated a differential representation of unclassified Erysipelotrichaceae (specifically over represented in PGM1), *Turicibacter* (highly prevalent in the Illumina runs), and potential *Ruminococcus* species. Taxa differentially represented by platform in the UPARSE pipelines included *Turicibacter*, unclassified Clostridiaceae, Clostridiales (highly prevalent in the PGM pipelines), *Eubacterium biforme*, *Lactobacillus* spp., unclassified Erysipelotrichaceae, *Ruminococcus*, *Anaerotruncus*, and Lachnospiraceae.

Further analysis using PhyloToAST [55] (Figure 6) showed that the *de novo* OTU picking approach in the QIIME pipeline identified the highest number of unique species (over 250) followed by the open reference OTU picking approach (approximately 180) and UPARSE (approximately 140). Relevant groups under detected by the open reference and UPARSE OTU picking approaches included *Bifidobacterium* spp. (absent in the open reference and UPARSE OTU picking approaches) and *Bifidobacterium adolescentis* (not identified by UPARSE). Differences in the detection profiles were also observed for lactobacilli. Bifidobacteria and lactobacilli are of significance for the samples analyzed in this study since these populations are expected to be impacted by prebiotic feeding, one of the conditions analyzed. Other taxa differentially identified by the different pipelines included species of *Tepidimicrobium, Thermacetogenium, Tindallia_Anoxynatronum,* and *Tissierella_Soehngenia.* These groups correspond to poorly characterized soil bacteria.

Finally, comparing the results of the pipelines intended to assess finer resolution features of the microbial communities, we found that the conclusions obtained in the previous analyses largely hold. For both pipelines, samples clustered in beta diversity PCoA plots according to treatment and sequencing run. Procrustes analysis showed that the primary difference in taxa assessments occurred due to the OTU/variant selection rather than the mechanism of assigning taxa to individual sequence representatives (Figure 7A). The number of OTUs or sequence representatives produced by DADA2 was smaller than the number of OTUs obtained from *de novo* OTU picking with at 99% similarity threshold (Figure 7B). At the genus level or lower, the pipeline using QIIME for both OTU picking and taxonomic assignment identified a total of 166 different taxa, while the pipeline using DADA2 for sequencing error suppression and taxonomic assignment detected 138 (Supplementary Table 3), with 65 groups identified at the species level. However, we observed that the taxonomy profiles determined by two different sequence representative selecting algorithms were similar and impacted mainly by the treatment. For the sequencing results produced by the Illumina MiSeq platform, both bioinformatic approaches support essentially the same biological conclusions.

**Discussion**

Next Generation Sequencing (NGS) has revolutionized the field of microbiome research [59-64]. Over the course of the last decade, NGS has become a faster, more accurate, and cost effective tool for the study of complex microbial communities [65]. In this study, with the objective of determining project-specific impacts of sequencing platforms and bioinformatics pipelines, we compared 16S rRNA amplicon sequencing data generated using three different platforms and 7 bioinformatics pipelines. GS FLX+, the traditional “gold standard” in terms of sequence length and quality prior to the development of Illumina and Life Sciences technologies, still generated the longest reads with the highest mean quality score per read after filtering, but this platform was not cost effective compared to newer platforms. Illumina MiSeq generated the highest number of reads per run but exhibited a lower mean quality score when compared to Roche GS FLX+. The Ion Torrent PGM platform yielded lower quality reads than MiSeq, and therefore fewer usable reads after quality filtering; however, performance was within the manufacturer’s parameters.

PCR-based high throughput amplicon sequencing introduces biases to the results obtained, regardless of the sequencing platform [16, 51]. In this study, Ion Torrent PGM sequencing was performed on amplicon libraries generated from three different PCR conditions (Figure 1). Modification of the PCR protocols for generation of sequencing libraries on the PGM platform directly impacted the number of identified species and sample diversity. Our results clearly indicate that modifications to the PCR conditions result in a differential representation of specific taxa. An increased number of PCR cycles probably increased amplification of background DNA and decreased specificity, as a lower annealing temperature. This is an important issue when analyzing low biomass samples, especially considering that DNA contamination of reagents and laboratory-grade water has been clearly demonstrated [66]. Other parameters that can impact taxa identification and relative quantification are DNA input and chosen amplification primers. The DNA input for the GS FLX+ and PGM standard protocols is 4 times greater than the input for amplicon generation for sequencing on the MiSeq instrument following the standard protocol. This could account for differences observed in the relative abundance of bacterial taxa since the less abundant bacterial DNA will have greater chances to be amplified when the amount of DNA is higher. No previous studies have compared DNA input on 16S rRNA sequencing data generation, although the DNA isolation procedure has been shown to have a significant impact on sequencing results [67]. Previous studies have shown an impact of the primer sets on 16S rRNA amplicon sequencing [26, 68]. In this study we evaluated the same set of primers for all amplifications (8F and 338R); however, a potential impact of adapters and barcodes specific to each platform added to the sequencing primers cannot be discarded. Moreover, primers targeting the V4 region of the 16S rRNA gene are currently the most widely used; however, our project initially used the 454 platform for amplicon sequencing analysis. Since the region V1-V2 was the most traditionally used across decades of research, and was reported optimal specifically for 16S rRNA amplicon pyrosequencing across a range of taxonomic classifications from phylum to family [69], we chose to target such region. We are aware, however, that recent studies provide support to targeting the V3-V4 regions of the 16S rRNA gene for studies of bacterial diversity and this is currently standard in our laboratory. We targeted the same region across platforms for the sake of comparison. Finally, standard library preparation protocols (GS-FLX+, PGM1, and MiSeq1/MiSeq2) coupled with the most widely used bioinformatics analysis procedure (QIIME2 = *de novo* OTU picking with removal of chimeras) showed a significant difference in PD between GS-FLX+ and PGM1, and in the number of identified species between the two MiSeq runs between PGM1 and MiSeq1.

Different sequencing platforms/pipelines generate results with differing phylogenetic distributions (Figure 5). As the sequencing chemistry differs between platforms, introducing internal biases, this finding is not without precedent [23-25, 70-72]. Furthermore, microbial genera often vary drastically in genome composition. For example, *Lactobacillus reuteri* has a GC content of 35.9%-38.9% [73] while *Bifidobacterium longum* has GC content of 60% [74]. This difference in genomic GC content impacts the ability of different platforms to accurately identify presence or absence, and to determine microbial abundance. As such, biases introduced as a result of the sequencing chemistry, and limitations of individual platforms to discriminate between microbes with varying genomic GC content makes it important to consider the technology used in relation to the scope of the project. Our study showed that UPARSE analysis yields fewer microbial genera and lower phylogenetic diversity than QIIME analysis (Figure 3A, Figure 6). Both UPARSE and QIIME pipelines start by quality-filtering reads, trimming them to a fixed length, optionally discarding singleton reads, and clustering the remaining reads. The differences between the two pipelines rely on the clustering algorithm. UPARSE uses a 'greedy' algorithm that performs chimera filtering and OTU clustering simultaneously, while QIIME performs chimera filtering as a separate step. In this study we evaluated both the *de novo* and open reference OTU picking in QIIME. The main advantage of the first method is that it does not require a collection of reference sequences before working with a new marker gene. The open-reference OTU picking combines closed-reference OTU picking, in which input sequences are aligned to pre-defined cluster centroids in a reference database, and if the input sequence does not match any reference sequence at a defined percent identity threshold, the sequence is excluded, and *de novo* OTU picking is applied to the remaining sequences. The OTU picking algorithm used by UPARSE does not require technology- or gene-specific parameters (such as an OTU size cutoff), algorithms (such as flowgram denoising) or data (such as a curated multiple alignment) [37]. The impact of different bioinformatics pipelines on the diversity captured from specific microbial ecosystems was investigated in Pylro et al. [57]. The study concluded that UniFrac distances between samples sequenced on both Illumina MiSeq and PGM were significantly correlated.

Procrustes analysis of data showed clear differences between data generated from each platform (Figure 4B and 7A). Nevertheless, despite differences in platform sequencing performance and data output, all three platforms were capable of discriminating samples by treatment and led us to similarly valid biological conclusions (Figure 4A). Our results are in agreement with a previous study that showed that soil samples analyzed by 16S rRNA sequencing using either the Illumina MiSeq or PGM platforms clustered by site rather than by sequencing platform [57].

Determining the most accurate and appropriate approaches to generate and analyze sequencing data from complex microbial communities remains an important goal of researchers focused on microbiota studies. Unfortunately without a standardized set of protocols from sample preparation and handling to final data analysis (and every step in between), it is impossible to eliminate biases from these studies entirely. However, making an effort to choose the correct sequencing platform for a given study as well as the most appropriate data analysis tools will drastically reduce errors in data acquisition and processing between studies. In this context, emerging analysis methods (methods that attempt to differentiate between sequences with similarity greater than the 97% convention) such as DADA2 [38] and Oligotyping [75] are an innovative and interesting new development in the processing of sequencing of microbial communities.  These methods differ from conventional methods in substantial ways, for example, the suppression of sequencing errors and estimation of their probability distribution. These matters are of fundamental importance to methods that attempt to differentiate biologically meaningful single nucleotide variation from sequencing data and warrant ongoing comparisons of relevant protocols and analysis methods for the study of complex microbial communities.

In our study, we compared DADA2 [38] and QIIME *de novo* OTU picking with a similarity threshold of 99% for OTU or variant calling, and then assignment of taxonomy was performed on each table using either the DADA2 taxonomy classification method and the QIIME taxonomy classification method. As with UPARSE, applying the DADA2 variant calling pipeline resulted in lower numbers of identified OTUs, resulting in significant differences between pipelines demonstrated by the Procrustes analysis. However, both pipelines resulted in clustering of samples by treatment and Illumina run. Moreover, a linear correlation analysis comparing relative abundances determined by the pipeline using QIIME for both OTU picking and taxonomic assignment and the pipeline using DADA2 for both sequencing error suppression and taxonomic assignment showed a Person’s r value of 0.93 indicating a strong correlation between values (Figure 7D). Although application of the DADA2 algorithm for taxonomy assignment resulted in a higher number of species identified, reliability on the assignment at sub genus level is largely unknown since this software has not been extensively tested and was developed with a mock community of known bacterial strains as opposed to be done on native communities. As such, use of this software for analysis of gut microbial communities should be done with caution. Traditional microbiology methods and whole genome shotgun sequencing data will confirm or deny whether single nucleotide polymorphisms of a single bacterial gene are sufficient to obtain reliable species level information within complex communities.

**Conclusion**

Our study confirmed differences between sequencing platforms and library preparation protocols in the identification of microbial diversity and species richness. Moreover, we showed that bioinformatics pipelines used to analyze sequence data yielded results that differ depending on specific parameters. However, we concluded that, specifically for our chicken-*Salmonella* infection study, despite these differences, samples from variable treatment groups were differentiated from one another regardless of the sequencing platform and/or bioinformatics pipeline used allowing us to draw similar conclusions. This suggested that the same biological conclusions could be drawn from data, as long as the data is collected and analyzed consistently throughout the course of the experiment. It is critical for researchers to take into consideration the limitations of each sequencing platform, and choose a system appropriate for their experimental design.

**List of abbreviations**

NGS Next Generation Sequencing

MBQC Microbiome Quality Control project

MAQC Microarray Quality Control project

QIIME Quantitative Insights Into Microbial Ecology

DADA2 Divisive Amplicon Denoising Algorithm2

ANOVA Analysis of variance

PD Phylogenetic Diversity

PCoA Principal Coordinate Analysis

ANOSIM Analysis of Similarities

PERMANOVA Permutational Multivariate Analysis of Variance

**Declarations**

**Ethics approval**

Animals were euthanized according to protocol #15-065-A, approved by the North Carolina State University Institutional Animal Care and Use Committee (OLAW# D16-00214) and sampled for gut microbiome analysis.

**Consent for publication**

Not applicable.

**Availability of data and materials**

This project is available at NCBI under BioProject ID PRJNA40025

**Competing interests**

The authors declare that they have no competing interests.

**Funding**

The Microbiome Core Facility is supported in part by the NIH/National Institute of Diabetes and Digestive and Kidney Diseases grant P30 DK34987. This research was also supported by the USDA-NIFA Award# 2012-68003-19621 and NCTraCs. I. A. is a Fulbright Scholar.

**Authors' contributions**

IA Performed bioinformatics and statistical analyses of data and wrote the manuscript.

JWA Contributed to manuscript writing and editing.

MBC and NB performed DNA isolation, barcoding, library preparation and sequencing of samples.

JR Performed bioinformatics analysis of data and contributed to manuscript writing and editing.

HH, MK Performed animal experiments and contributed to manuscript writing and editing.

AB, MM, and RA performed animal experiments.

MAAP Designed the experiments, advised IA, contributed to bioinformatics and statistical analyses of data and wrote the manuscript.

All authors read and approved the final manuscript.

**Acknowledgements**

We are grateful to Dr. J. Bruno-Barcena and Mr. Hunter Whittington for their input on data analysis using Phylotoast and iTOL tools.

**References**

1. Thompson AL, Monteagudo-Mera A, Cadenas MB, Lampl ML, Azcarate-Peril MA: **Milk- and solid-feeding practices and daycare attendance are associated with differences in bacterial diversity, predominant communities, and metabolic and immune function of the infant gut microbiome**. *Frontiers in cellular and infection microbiology* 2015, **5**:3.

2. Turnbaugh PJ, Ridaura VK, Faith JJ, Rey FE, Knight R, Gordon JI: **The effect of diet on the human gut microbiome: a metagenomic analysis in humanized gnotobiotic mice**. *Sci Transl Med* 2009, **1**(6):6ra14.

3. Hildebrandt MA, Hoffmann C, Sherrill-Mix SA, Keilbaugh SA, Hamady M, Chen YY, Knight R, Ahima RS, Bushman F, Wu GD: **High-fat diet determines the composition of the murine gut microbiome independently of obesity**. *Gastroenterology* 2009, **137**(5):1716-1724 e1711-1712.

4. Bonder MJ, Tigchelaar EF, Cai X, Trynka G, Cenit MC, Hrdlickova B, Zhong H, Vatanen T, Gevers D, Wijmenga C *et al*: **The influence of a short-term gluten-free diet on the human gut microbiome**. *Genome Med* 2016, **8**(1):45.

5. Sommer F, Nookaew I, Sommer N, Fogelstrand P, Bäckhed F: **Site-specific programming of the host epithelial transcriptome by the gut microbiota**. *Genome biology* 2015, **16**(1).

6. Drumo R, Pesciaroli M, Ruggeri J, Tarantino M, Chirullo B, Pistoia C, Petrucci P, Martinelli N, Moscati L, Manuali E *et al*: **Salmonella enterica Serovar Typhimurium Exploits Inflammation to Modify Swine Intestinal Microbiota**. *Frontiers in cellular and infection microbiology* 2015, **5**.

7. Kim HJ, Li H, Collins JJ, Ingber DE: **Contributions of microbiome and mechanical deformation to intestinal bacterial overgrowth and inflammation in a human gut-on-a-chip**. *Proceedings of the National Academy of Sciences of the United States of America* 2016, **113**(1):E7-e15.

8. Candon S, Perez-Arroyo A, Marquet C, Valette F, Foray AP, Pelletier B, Milani C, Ventura M, Bach JF, Chatenoud L: **Antibiotics in Early Life Alter the Gut Microbiome and Increase Disease Incidence in a Spontaneous Mouse Model of Autoimmune Insulin-Dependent Diabetes**. *PloS one* 2015, **10**(5).

9. Vandenplas Y, Huys G, Daube G: **Probiotics: an update**. *Jornal de pediatria* 2015, **91**(1):6-21.

10. Gao Z, Guo B, Gao R, Zhu Q, Wu W, Qin H: **Probiotics modify human intestinal mucosa-associated microbiota in patients with colorectal cancer**. *Molecular medicine reports* 2015.

11. Nami Y, Haghshenas B, Abdullah N, Barzegari A, Radiah D, Rosli R, Khosroushahi AY: **Probiotics or antibiotics: future challenges in medicine**. *Journal of medical microbiology* 2015, **64**(Pt 2):137-146.

12. Vandenplas Y, Zakharova I, Dmitrieva Y: **Oligosaccharides in infant formula: more evidence to validate the role of prebiotics**. *The British journal of nutrition* 2015, **113**(9):1339-1344.

13. Monteagudo-Mera A, Arthur JC, Jobin C, Keku T, Bruno-Barcena JM, Azcarate-Peril MA: **High purity galacto-oligosaccharides enhance specific Bifidobacterium species and their metabolic activity in the mouse gut microbiome**. *Beneficial microbes* 2016:1-18.

14. Maurice CF, Haiser HJ, Turnbaugh PJ: **Xenobiotics shape the physiology and gene expression of the active human gut microbiome**. *Cell* 2013, **152**(1-2):39-50.

15. Woo PC, Lau SK, Teng JL, Tse H, Yuen KY: **Then and now: use of 16S rDNA gene sequencing for bacterial identification and discovery of novel bacteria in clinical microbiology laboratories**. *Clin Microbiol Infect* 2008, **14**(10):908-934.

16. Schloss PD, Gevers D, Westcott SL: **Reducing the effects of PCR amplification and sequencing artifacts on 16S rRNA-based studies**. *PLoS One* 2011, **6**(12):e27310.

17. Quail MA, Smith M, Coupland P, Otto TD, Harris SR, Connor TR, Bertoni A, Swerdlow HP, Gu Y: **A tale of three next generation sequencing platforms: comparison of Ion Torrent, Pacific Biosciences and Illumina MiSeq sequencers**. *BMC genomics* 2012, **13**:341.

18. Luo C, Tsementzi D, Kyrpides N, Read T, Konstantinidis KT: **Direct comparisons of Illumina vs. Roche 454 sequencing technologies on the same microbial community DNA sample**. *PLoS One* 2012, **7**(2):e30087.

19. Li X, Buckton AJ, Wilkinson SL, John S, Walsh R, Novotny T, Valaskova I, Gupta M, Game L, Barton PJ *et al*: **Towards clinical molecular diagnosis of inherited cardiac conditions: a comparison of bench-top genome DNA sequencers**. *PLoS One* 2013, **8**(7):e67744.

20. Claesson MJ, Wang Q, O'Sullivan O, Greene-Diniz R, Cole JR, Ross RP, O'Toole PW: **Comparison of two next-generation sequencing technologies for resolving highly complex microbiota composition using tandem variable 16S rRNA gene regions**. *Nucleic acids research* 2010, **38**(22):e200.

21. Salipante SJ, Kawashima T, Rosenthal C, Hoogestraat DR, Cummings LA, Sengupta DJ, Harkins TT, Cookson BT, Hoffman NG: **Performance Comparison of Illumina and Ion Torrent Next-Generation Sequencing Platforms for 16S rRNA-Based Bacterial Community Profiling**. *Applied and environmental microbiology* 2014, **80**(24):7583-7591.

22. Tremblay J, Singh K, Fern A, Kirton ES, He S, Woyke T, Lee J, Chen F, Dangl JL, Tringe SG: **Primer and platform effects on 16S rRNA tag sequencing**. *Front Microbiol* 2015, **6**:771.

23. Jones MB, Highlander SK, Anderson EL, Li W, Dayrit M, Klitgord N, Fabani MM, Seguritan V, Green J, Pride DT *et al*: **Library preparation methodology can influence genomic and functional predictions in human microbiome research**. *Proc Natl Acad Sci U S A* 2015, **112**(45):14024-14029.

24. Yu G, Fadrosh D, Goedert JJ, Ravel J, Goldstein AM: **Nested PCR Biases in Interpreting Microbial Community Structure in 16S rRNA Gene Sequence Datasets**. *PLoS One* 2015, **10**(7):e0132253.

25. Dechesne A, Musovic S, Palomo A, Diwan V, Smets BF: **Underestimation of ammonia-oxidizing bacteria abundance by amplification bias in amoA-targeted qPCR**. *Microbial biotechnology* 2016.

26. Sinha R, Abnet CC, White O, Knight R, Huttenhower C: **The microbiome quality control project: baseline study design and future directions**. *Genome Biol* 2015, **16**:276.

27. Liu L, Li Y, Li S, Hu N, He Y, Pong R, Lin D, Lu L, Law M: **Comparison of next-generation sequencing systems**. *Journal of biomedicine & biotechnology* 2012, **2012**:251364.

28. Shokralla S, Spall JL, Gibson JF, Hajibabaei M: **Next-generation sequencing technologies for environmental DNA research**. *Molecular ecology* 2012, **21**(8):1794-1805.

29. Whiteley AS, Jenkins S, Waite I, Kresoje N, Payne H, Mullan B, Allcock R, O'Donnell A: **Microbial 16S rRNA Ion Tag and community metagenome sequencing using the Ion Torrent (PGM) Platform**. *J Microbiol Methods* 2012, **91**(1):80-88.

30. Indugu N, Bittinger K, Kumar S, Vecchiarelli B, Pitta D: **A comparison of rumen microbial profiles in dairy cows as retrieved by 454 Roche and Ion Torrent (PGM) sequencing platforms**. *PeerJ* 2016, **4**:e1599.

31. Sinclair L, Osman OA, Bertilsson S, Eiler A: **Microbial community composition and diversity via 16S rRNA gene amplicons: evaluating the illumina platform**. *PLoS One* 2015, **10**(2):e0116955.

32. Ferrarini M, Moretto M, Ward JA, Surbanovski N, Stevanovic V, Giongo L, Viola R, Cavalieri D, Velasco R, Cestaro A *et al*: **An evaluation of the PacBio RS platform for sequencing and de novo assembly of a chloroplast genome**. *BMC genomics* 2013, **14**:670.

33. Castelino M, Eyre S, Moat J, Fox G, Martin P, Ijaz U, Quince C, Ho P, Upton M, Barton A: **The skin microbiome in psoriatic arthritis: methodology development and pilot data**. *Lancet* 2015, **385 Suppl 1**:S27.

34. Shendure J, Ji H: **Next-generation DNA sequencing**. *Nat Biotechnol* 2008, **26**(10):1135-1145.

35. Loman NJ, Misra RV, Dallman TJ, Constantinidou C, Gharbia SE, Wain J, Pallen MJ: **Performance comparison of benchtop high-throughput sequencing platforms**. *Nat Biotechnol* 2012, **30**(5):434-439.

36. Caporaso JG, Kuczynski J, Stombaugh J, Bittinger K, Bushman FD, Costello EK, Fierer N, Pena AG, Goodrich JK, Gordon JI *et al*: **QIIME allows analysis of high-throughput community sequencing data**. *Nat Methods* 2010, **7**(5):335-336.

37. Edgar RC: **UPARSE: highly accurate OTU sequences from microbial amplicon reads**. *Nature methods* 2013, **10**(10):996-998.

38. Callahan BJ, McMurdie PJ, Rosen MJ, Han AW, Johnson AJ, Holmes SP: **DADA2: High-resolution sample inference from Illumina amplicon data**. *Nat Methods* 2016, **13**(7):581-583.

39. Azcarate-Peril MA, Ritter AJ, Savaiano D, Monteagudo-Mera A, Anderson C, Magness ST, Klaenhammer TR: **Impact of short-chain galactooligosaccharides on the gut microbiome of lactose-intolerant individuals**. *Proc Natl Acad Sci U S A* 2017, **114**(3):E367-E375.

40. Haas BJ, Gevers D, Earl AM, Feldgarden M, Ward DV, Giannoukos G, Ciulla D, Tabbaa D, Highlander SK, Sodergren E *et al*: **Chimeric 16S rRNA sequence formation and detection in Sanger and 454-pyrosequenced PCR amplicons**. *Genome Res* 2011, **21**(3):494-504.

41. Edgar RC, Haas BJ, Clemente JC, Quince C, Knight R: **UCHIME improves sensitivity and speed of chimera detection**. *Bioinformatics* 2011, **27**(16):2194-2200.

42. Langille MG, Zaneveld J, Caporaso JG, McDonald D, Knights D, Reyes JA, Clemente JC, Burkepile DE, Vega Thurber RL, Knight R *et al*: **Predictive functional profiling of microbial communities using 16S rRNA marker gene sequences**. *Nat Biotechnol* 2013, **31**(9):814-821.

43. Devine AA, Gonzalez A, Speck KE, Knight R, Helmrath M, Lund PK, Azcarate-Peril MA: **Impact of ileocecal resection and concomitant antibiotics on the microbiome of the murine jejunum and colon**. *PLoS One* 2013, **8**(8):e73140.

44. Edwards U, Rogall T, Blocker H, Emde M, Bottger EC: **Isolation and direct complete nucleotide determination of entire genes. Characterization of a gene coding for 16S ribosomal RNA**. *Nucleic Acids Res* 1989, **17**(19):7843-7853.

45. Fierer N, Hamady M, Lauber CL, Knight R: **The influence of sex, handedness, and washing on the diversity of hand surface bacteria**. *Proc Natl Acad Sci U S A* 2008, **105**(46):17994-17999.

46. GS Data Analysis Software. Roche Applied Science. Indianapolis, IN, USA.

47. Ion Personal Genome Machine. Life Technologies. Grand Island, NY, USA.

48. CASAVA 1.8.2. Illumina, Inc. San Diego, CA, USA.

49. Aronesty E: **Comparison of Sequencing Utility Programs**. *The Open Bioinformatics Journal* 2013, **7**:1-8.

50. Edgar RC: **Search and clustering orders of magnitude faster than BLAST**. *Bioinformatics* 2010, **26**(19):2460-2461.

51. Haas BJ, Gevers D, Earl AM, Feldgarden M, Ward DV, Giannoukos G, Ciulla D, Tabbaa D, Highlander SK, Sodergren E *et al*: **Chimeric 16S rRNA sequence formation and detection in Sanger and 454-pyrosequenced PCR amplicons**. *Genome research* 2011, **21**(3):494-504.

52. Price MN, Dehal PS, Arkin AP: **FastTree 2--approximately maximum-likelihood trees for large alignments**. *PLoS One* 2010, **5**(3):e9490.

53. Lozupone C, Hamady M, Knight R: **UniFrac--an online tool for comparing microbial community diversity in a phylogenetic context**. *BMC Bioinformatics* 2006, **7**:371.

54. Lozupone C, Knight R: **UniFrac: a new phylogenetic method for comparing microbial communities**. *Appl Environ Microbiol* 2005, **71**(12):8228-8235.

55. Dabdoub SM, Fellows ML, Paropkari AD, Mason MR, Huja SS, Tsigarida AA, Kumar PS: **PhyloToAST: Bioinformatics tools for species-level analysis and visualization of complex microbial datasets**. *Sci Rep* 2016, **6**:29123.

56. Letunic I, Bork P: **Interactive tree of life (iTOL) v3: an online tool for the display and annotation of phylogenetic and other trees**. *Nucleic Acids Res* 2016, **44**(W1):W242-W245.

57. Pylro VS, Roesch LF, Morais DK, Clark IM, Hirsch PR, Totola MR: **Data analysis for 16S microbial profiling from different benchtop sequencing platforms**. *J Microbiol Methods* 2014, **107**:30-37.

58. Eeckhaut V, Machiels K, Perrier C, Romero C, Maes S, Flahou B, Steppe M, Haesebrouck F, Sas B, Ducatelle R *et al*: **Butyricicoccus pullicaecorum in inflammatory bowel disease**. *Gut* 2013, **62**(12):1745-1752.

59. Nekrutenko A, Taylor J: **Next-generation sequencing data interpretation: enhancing reproducibility and accessibility**. *Nature reviews Genetics* 2012, **13**(9):667-672.

60. Metzker ML: **Emerging technologies in DNA sequencing**. *Genome research* 2005, **15**(12):1767-1776.

61. Metzker ML: **Sequencing technologies - the next generation**. *Nature reviews Genetics* 2010, **11**(1):31-46.

62. Zhang J, Chiodini R, Badr A, Zhang G: **The impact of next-generation sequencing on genomics**. *Journal of genetics and genomics = Yi chuan xue bao* 2011, **38**(3):95-109.

63. Meldrum C, Doyle MA, Tothill RW: **Next-generation sequencing for cancer diagnostics: a practical perspective**. *The Clinical biochemist Reviews / Australian Association of Clinical Biochemists* 2011, **32**(4):177-195.

64. Wu K, Huang RS, House L, Cho WC: **Next-generation sequencing for lung cancer**. *Future oncology* 2013, **9**(9):1323-1336.

65. Venter JC, Levy S, Stockwell T, Remington K, Halpern A: **Massive parallelism, randomness and genomic advances**. *Nature genetics* 2003, **33 Suppl**:219-227.

66. Salter SJ, Cox MJ, Turek EM, Calus ST, Cookson WO, Moffatt MF, Turner P, Parkhill J, Loman NJ, Walker AW: **Reagent and laboratory contamination can critically impact sequence-based microbiome analyses**. *BMC Biol* 2014, **12**:87.

67. Kennedy NA, Walker AW, Berry SH, Duncan SH, Farquarson FM, Louis P, Thomson JM, Consortium UIG, Satsangi J, Flint HJ *et al*: **The impact of different DNA extraction kits and laboratories upon the assessment of human gut microbiota composition by 16S rRNA gene sequencing**. *PLoS One* 2014, **9**(2):e88982.

68. Fouhy F, Clooney AG, Stanton C, Claesson MJ, Cotter PD: **16S rRNA gene sequencing of mock microbial populations- impact of DNA extraction method, primer choice and sequencing platform**. *BMC Microbiol* 2016, **16**(1):123.

69. Hamp TJ, Jones WJ, Fodor AA: **Effects of experimental choices and analysis noise on surveys of the "rare biosphere"**. *Appl Environ Microbiol* 2009, **75**(10):3263-3270.

70. Ibarbalz FM, Perez MV, Figuerola EL, Erijman L: **The bias associated with amplicon sequencing does not affect the quantitative assessment of bacterial community dynamics**. *PLoS One* 2014, **9**(6):e99722.

71. Kennedy K, Hall MW, Lynch MD, Moreno-Hagelsieb G, Neufeld JD: **Evaluating bias of illumina-based bacterial 16S rRNA gene profiles**. *Applied and environmental microbiology* 2014, **80**(18):5717-5722.

72. Schirmer M, Ijaz UZ, D'Amore R, Hall N, Sloan WT, Quince C: **Insight into biases and sequencing errors for amplicon sequencing with the Illumina MiSeq platform**. *Nucleic acids research* 2015, **43**(6):e37.

73. Mendes-Soares H, Suzuki H, Hickey RJ, Forney LJ: **Comparative functional genomics of Lactobacillus spp. reveals possible mechanisms for specialization of vaginal lactobacilli to their environment**. *Journal of bacteriology* 2014, **196**(7):1458-1470.

74. Schell MA, Karmirantzou M, Snel B, Vilanova D, Berger B, Pessi G, Zwahlen MC, Desiere F, Bork P, Delley M *et al*: **The genome sequence of Bifidobacterium longum reflects its adaptation to the human gastrointestinal tract**. *Proc Natl Acad Sci U S A* 2002, **99**(22):14422-14427.

75. Eren AM, Maignien L, Sul WJ, Murphy LG, Grim SL, Morrison HG, Sogin ML: **Oligotyping: Differentiating between closely related microbial taxa using 16S rRNA gene data**. *Methods Ecol Evol* 2013, **4**(12).

**Figure Legends**

**Figure 1.** Schematic of the experimental design of this study to test impact of library preparation methods and protocols on diversity and relative abundance of bacteria. Protocol steps are indicated on the left. Standard methods are in black boxes while non-standard methods with modified conditions are shown in grey boxes.

**Figure 2.** Evaluated bioinformatics pipelines using QIIME[36] and UPARSE[37] using two different OTU picking methods (QIIME only) either with or without chimera removal steps.

**Figure 3.** A comparison of phylogenetic diversity (PD) and species richness (S) between the 6 runs (GS FLX, MiSeq1, MiSeq2, PGM1, PGM 2 and PGM3) and in each pipeline (A) Phylogenetic diversity (B) Species Richness. Panels on the right show a matrix comparison between pipelines. Numbers within cells indicate *P*-values >0.05 <0.1. **P* <0.01,***P* <0.001

**Figure 4.** A) Principal Coordinates Analysis PCoA (Unweighted UniFrac) plots of data generated by the three different platforms, analyzed by different bioinformatics pipelines and colored according to treatment group (Prebiotics, control and *Salmonella*-vaccinated). PERMANOVA F and *P* values and ANOSIM R and *P* values are indicated. B) Procrustes analysis of sequencing data from the different platforms analyzed with the QIIME2 (*de novo* OTU picking plus chimera depletion). M and *P* values are indicated in the figure.

**Figure 5.** Selected differences in relative abundances of the most impacted taxa according to data generated by different platforms (indicated by different colors) and bioniformatic analysis pipelines (indicated across the top). The full figure can be seen in Supplementary Figure 2.

**Figure 6.** Unique species identified by the different bioinformatic analysis schemes. Boxes indicate taxa not detected by open reference OTU picking (QIIME) and UPARSE methods, which may be of significance for the study.

**Figure 7.** Comparisons made between the two different OTU/variant calling (either DADA2 or QIIME *de novo* OTU picking at 99% similarity) and the two different taxa assigment algorithms (DADA2 or QIIME using the Greengenes database). Labels are: QIIME.QIIME indicating QIIME was used for OTU picking and taxonomic assigment, QIIME.DADA2 indicating QIIME was used for OTU picking and DADA2 was used for taxonomic assignment, DADA2.QIIME indicating the DADA2 was used for sequencing error supression and QIIME was used for taxonomic assignment, and DADA2.DADA2 indicating that used for both sequencing error suppresion and taxonomic assignment. (A) Procrustes analysis. (B) A comparison of the number of OTUs identified by DADA2 (clear boxes) and QIIME *de novo* OTU picking at 99% similarity (shaded boxes). (C) Taxonomic profiles of samples grouped by treatment and bioinformatics pipeline. Only major taxa are indicated in the Figure. (D) Correlation analysis of relative abundances of bacterial taxa at species level. For a complete list see Supplementary Table 3.

**Table 1.** Primer sequence information for all platforms.

| **Platform** | **Primer Name** | **Sequence (5’ -3’)** | **Targeting Region** |
| --- | --- | --- | --- |
| Roche 454 | Roche Titanium Fusion Primer A | CCATCTCATCCCTGCGTGTCTCCGACTCAG | V1-V2 |
|  | Universal Bacterial Primer 8F | AGAGTTTGATCCTGGCTCAG | V1-V2 |
|  | Roche Titanium Primer B | CCTATCCCCTGTGTGCCTTGGCAGTCTCAG | V1-V2 |
|  | Reverse Bacterial Primer 338R | GCTGCCTCCCGTAGGAGT | V1-V2 |
| Illumina MiSeq | Forward Primer | TCGTCGGCAGCGTCAGATGTGTATAAGAGACAGAGAGTTTGATCCTGGCTCAG | V1-V2 |
|  | Reverse Primer | GTCTCGTGGGCTCGGAGATGTGTATAAGAGACAGGCTGCCTCCCGTAGGAGT | V1-V2 |
| Ion Torrent PGM | Forward Primer composed of Ion Torrent adapter A | CCATCTCATCCCTGCGTGTCTCCGACTCAG | V1-V2 |
|  | Universal Bacterial Primer 8F | AGAGTTTGATCCTGGCTCAG | V1-V2 |
|  | Reverse Primer of Ion Torrent trP1 adapter | CCTCTCTATGGGCAGTCGGTGAT | V1-V2 |
|  | Reverse Bacterial Primer 338R | GCTGCCTCCCGTAGGAGT | V1-V2 |

| Sample | Intestinal Location | Treatment | Chicken | *Salmonella*-challenged | Time point (week) |
| --- | --- | --- | --- | --- | --- |
| M848A | Cecum | Control | 7 | Yes | 7 |
| M480A | Cecum | Vaccinated | 1 | No | 6 |
| M736A | Cecum | Vaccinated | 2 | No | 9 |
| M572A | Cecum | Vaccinated | 3 | No | 7 |
| M576A | Cecum | Vaccinated | 4 | No | 7 |
| M988A | Cecum | Vaccinated | 2 | Yes | 8 |
| M908A | Cecum | Vaccinated | 3 | Yes | 7 |
| M368A | Cecum | Prebiotics | 1 | No | 5 |
| M452A | Cecum | Prebiotics | 1 | No | 6 |
| M620A | Cecum | Prebiotics | 1 | No | 8 |
| M704A | Cecum | Prebiotics | 1 | No | 9 |
| M540A | Cecum | Prebiotics | 2 | No | 7 |
| M464A | Cecum | Prebiotics | 4 | No | 6 |
| M884A | Cecum | Prebiotics | 4 | Yes | 7 |

**Table 2.** Characteristics of samples used in this study.

**Table 3.** GS FLX (Roche), PGM (Ion Torrent, Life Technologies) and MiSeq (Illumina) platform comparison. Data was obtained from the corresponding platform’s website.

|  | Roche 454 | Ion Torrent | Illumina MiSeq |
| --- | --- | --- | --- |
| Sequencing Kit | GS FLX Titanium XLR70 | PGM 400 Sequencing | MiSeq Reagent Kits v2 |
| Expected Read Length | Up to 600 bp | Up to 400 bp | MiSeq Reagent Kit v2: Up to 2 x 250 bp |
| Typical Throughput | 450 Mb | Ion 314™ Chip v2: Up to 100 Mb  Ion 316™ Chip v2: Up to 1 Gb  Ion 318™ Chip v2: Up to 2 Gb | Up to 8.5 Gb |
| Reads per Run | ~1,000,000 shotgun,  ~700,000 amplicon | Ion 314™ Chip v2: 400-550 thousand  Ion 316™ Chip v2: 2-3 millions  Ion 318™ Chip v2: 4-5.5 millions | ~15 million reads |
| Consensus Accuracy | 99.995% | 99% | 99% |
| Run Time | 10 hours | Ion 314™ Chip v2: 2.3  to 3.7 hours  Ion 316™ Chip v2: 3.0  to 4.9 hours  Ion 318™ Chip v2: 4.4  to 7.3 hours | 4 hours and  approximately 39 hours  depending on the  number of cycles |
| Sample Input | gDNA, cDNA, or amplicons (PCR products) | gDNA, cDNA, or amplicons (PCR products) | gDNA, cDNA, or amplicons (PCR products)  Small genome, amplicon, and targeted gene panel sequencing |
| Weight | 532 lbs (242 kg) | 65 lbs (30 kg) | 120 lbs (54.5 kg) |
| Instrument cost | ~$500 K | ~ $80k | ~ $125k |

**Table 4.** Comparative summary of sequencing depth, reads length, and quality between GS FLEX, PGM, and MiSeq platforms.

| Platform | Raw reads | Filtered reads* | Mean Length after filtering (bp) | Percentage of reads kept after filtering | Mean quality score before quality filtering | Number of identified OTUs |
| --- | --- | --- | --- | --- | --- | --- |
| Roche 454 | 118,018 | 113,306 | 377 | 96% | 37.7 | 1,028 |
| Ion Torrent PGM | 481,593 | 71,652 | 297 | 14.9% | 23.4 | 2,747 |
| Illumina Miseq | 4,149,441 | 3,811,042 | 334 | 91.8% | 37.5 | 3,731 |

* Number of reads after filtering, reads of less than 200 nucleotides and with quality scores below 25 were removed

**Supplementary figures legends**

**Supplementary Figure S1.** Principal Coordinates Analysis PCoA (Unweighted UniFrac) plots of data generated by the three different platforms, analyzed by different bioinformatics pipelines and colored according to sequencing platform. PERMANOVA F and *P* values and ANOSIM R and *P* values are indicated.

**Supplementary Figure S2.** Differences in relative abundances of the most impacted taxa according to data generated by different platforms (indicated by different colors) and bioniformatic analysis pipelines (indicated across the top).
