# Supplementary material for: A comparison of sequencing platforms and bioinformatics pipelines for compositional analysis of the gut microbiome
Source: BMC Microbiol. 2017 Sep 13;17:194. doi: 10.1186/s12866-017-1101-8 (PMC5598039; doi:10.1186/s12866-017-1101-8)
Supplement: Supplementary file 2 — Figure S1. Principal Coordinates Analysis PCoA (Unweighted UniFrac) plots of data generated by the three different platforms, analyzed by different bioinformatics pipelines and colored according to sequencing platform. PERMANOVA F and P values and ANOSIM R and P values are indicated. Figure S2. Differences in relative abundances of the most impacted taxa according to data generated by different platforms (indicated by different colors) and bioniformatic analysis pipelines (indicated across the top). (PPTX 4115 kb) [file 12866_2017_1101_MOESM2_ESM.pptx]

## Slide 1
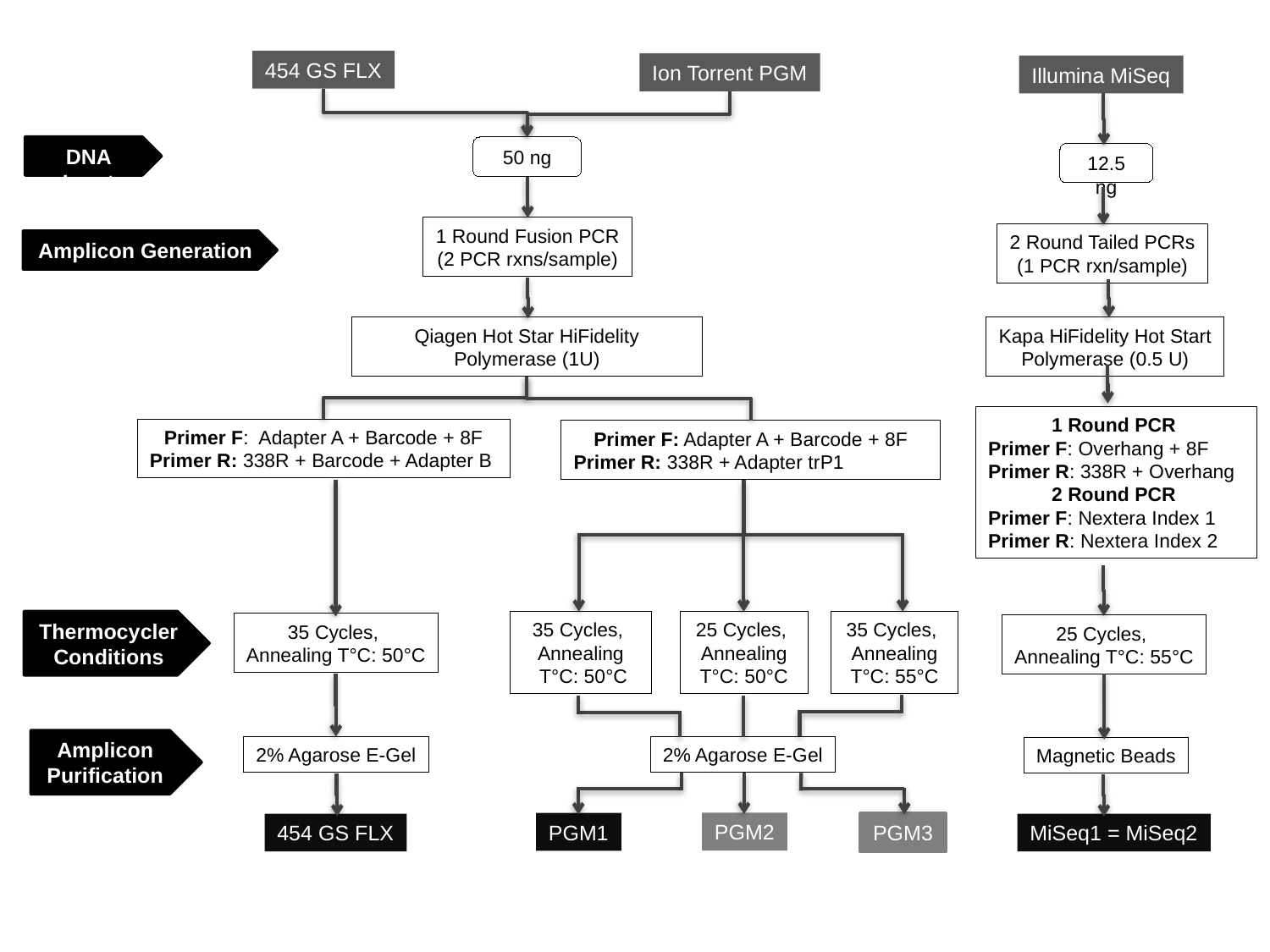

454 GS FLX
Ion Torrent PGM
Illumina MiSeq
DNA Input
50 ng
12.5 ng
1 Round Fusion PCR
(2 PCR rxns/sample)
2 Round Tailed PCRs
(1 PCR rxn/sample)
Amplicon Generation
Qiagen Hot Star HiFidelity Polymerase (1U)
Kapa HiFidelity Hot Start
Polymerase (0.5 U)
1 Round PCR
Primer F: Overhang + 8F
Primer R: 338R + Overhang
2 Round PCR
Primer F: Nextera Index 1
Primer R: Nextera Index 2
Primer F: Adapter A + Barcode + 8F
Primer R: 338R + Barcode + Adapter B
Primer F: Adapter A + Barcode + 8F
Primer R: 338R + Adapter trP1
35 Cycles,
Annealing
 T°C: 50°C
25 Cycles,
Annealing T°C: 50°C
35 Cycles,
Annealing T°C: 55°C
Thermocycler
Conditions
35 Cycles,
Annealing T°C: 50°C
25 Cycles,
Annealing T°C: 55°C
Amplicon
Purification
2% Agarose E-Gel
2% Agarose E-Gel
Magnetic Beads
PGM2
PGM1
454 GS FLX
PGM3
MiSeq1 = MiSeq2

## Slide 2
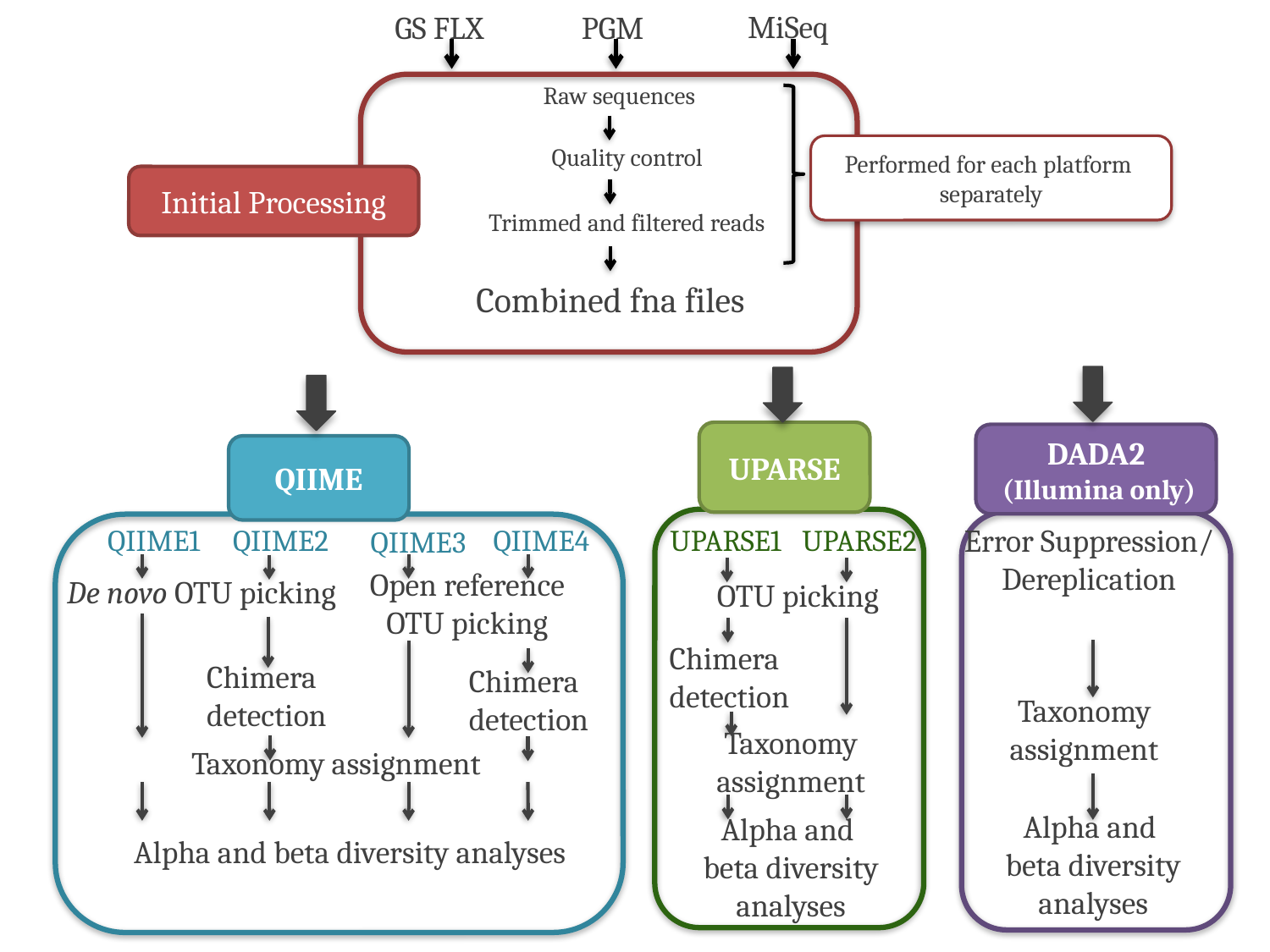

MiSeq
PGM
GS FLX
Raw sequences
Quality control
Performed for each platform
separately
Initial Processing
Trimmed and filtered reads
Combined fna files
UPARSE
DADA2
 (Illumina only)
QIIME
Error Suppression/ Dereplication
QIIME1
QIIME2
UPARSE1
UPARSE2
QIIME4
QIIME3
Open reference
OTU picking
De novo OTU picking
OTU picking
Chimera
detection
Chimera
detection
Chimera
detection
Taxonomy
assignment
Taxonomy
assignment
Taxonomy assignment
Alpha and
beta diversity analyses
Alpha and
beta diversity analyses
Alpha and beta diversity analyses

## Slide 3
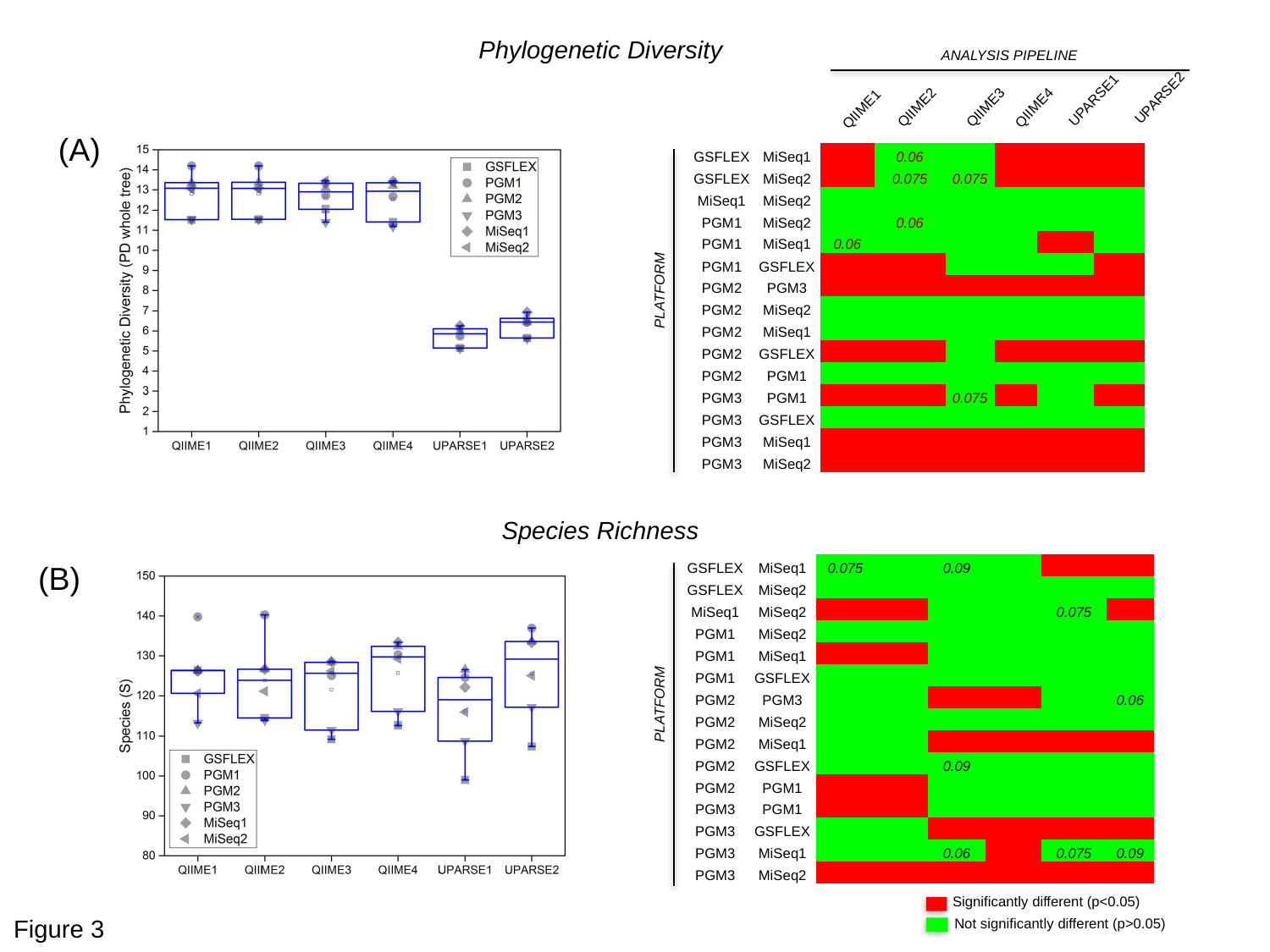

Phylogenetic Diversity
ANALYSIS PIPELINE
UPARSE2
UPARSE1
QIIME3
QIIME2
QIIME4
QIIME1
(A)
| GSFLEX | MiSeq1 | 0.015 | 0.06 | 0.18 | 0.015 | 0.015 | 0.015 |
| --- | --- | --- | --- | --- | --- | --- | --- |
| GSFLEX | MiSeq2 | 0.03 | 0.075 | 0.075 | 0.03 | 0.015 | 0.03 |
| MiSeq1 | MiSeq2 | 1 | 1 | 1 | 1 | 1 | 1 |
| PGM1 | MiSeq2 | 0.135 | 0.06 | 0.69 | 1 | 0.555 | 1 |
| PGM1 | MiSeq1 | 0.06 | 0.12 | 1 | 0.645 | 0.045 | 0.105 |
| PGM1 | GSFLEX | 0.015 | 0.015 | 1 | 0.225 | 0.195 | 0.015 |
| PGM2 | PGM3 | 0.015 | 0.03 | 0.015 | | 0.015 | 0.015 |
| PGM2 | MiSeq2 | 1 | 1 | 1 | 1 | 1 | 1 |
| PGM2 | MiSeq1 | 1 | 1 | 1 | 1 | 1 | 0.15 |
| PGM2 | GSFLEX | 0.015 | 0.015 | 0.33 | 0.03 | 0.015 | 0.015 |
| PGM2 | PGM1 | 0.36 | 0.435 | 1 | 1 | 1 | 1 |
| PGM3 | PGM1 | 0.015 | 0.015 | 0.075 | 0.015 | 0.105 | 0.015 |
| PGM3 | GSFLEX | 1 | 1 | 1 | 1 | 1 | 1 |
| PGM3 | MiSeq1 | 0.015 | 0.015 | 0.015 | 0.015 | 0.015 | 0.015 |
| PGM3 | MiSeq2 | 0.045 | 0.03 | 0.015 | 0.015 | 0.015 | 0.015 |
PLATFORM
Species Richness
(B)
| GSFLEX | MiSeq1 | 0.075 | 0.195 | 0.09 | 0.105 | 0.03 | 0.03 |
| --- | --- | --- | --- | --- | --- | --- | --- |
| GSFLEX | MiSeq2 | 1 | 1 | 1 | 1 | 0.36 | 0.93 |
| MiSeq1 | MiSeq2 | 0.015 | 0.015 | 0.105 | 0.225 | 0.075 | 0.015 |
| PGM1 | MiSeq2 | 1 | 1 | 1 | 1 | 1 | 1 |
| PGM1 | MiSeq1 | 0.015 | 0.015 | 1 | 1 | 0.705 | 0.195 |
| PGM1 | GSFLEX | 1 | 1 | 1 | 1 | 1 | 1 |
| PGM2 | PGM3 | 0.18 | 0.165 | 0.03 | 0.03 | 0.105 | 0.06 |
| PGM2 | MiSeq2 | 1 | 1 | 1 | 1 | 1 | 1 |
| PGM2 | MiSeq1 | 0.255 | 0.255 | 0.015 | 0.015 | 0.015 | 0.015 |
| PGM2 | GSFLEX | 1 | 1 | 0.09 | 0.285 | 1 | 1 |
| PGM2 | PGM1 | 0.015 | 0.03 | 1 | 1 | 1 | 1 |
| PGM3 | PGM1 | 0.015 | 0.03 | 1 | 1 | 1 | 1 |
| PGM3 | GSFLEX | 0.3 | 0.225 | 0.015 | 0.015 | 0.015 | 0.015 |
| PGM3 | MiSeq1 | 1 | 1 | 0.06 | 0.03 | 0.075 | 0.09 |
| PGM3 | MiSeq2 | 0.015 | 0.015 | 0.015 | 0.03 | 0.015 | 0.015 |
PLATFORM
Significantly different (p<0.05)
Figure 3
Not significantly different (p>0.05)

## Slide 4
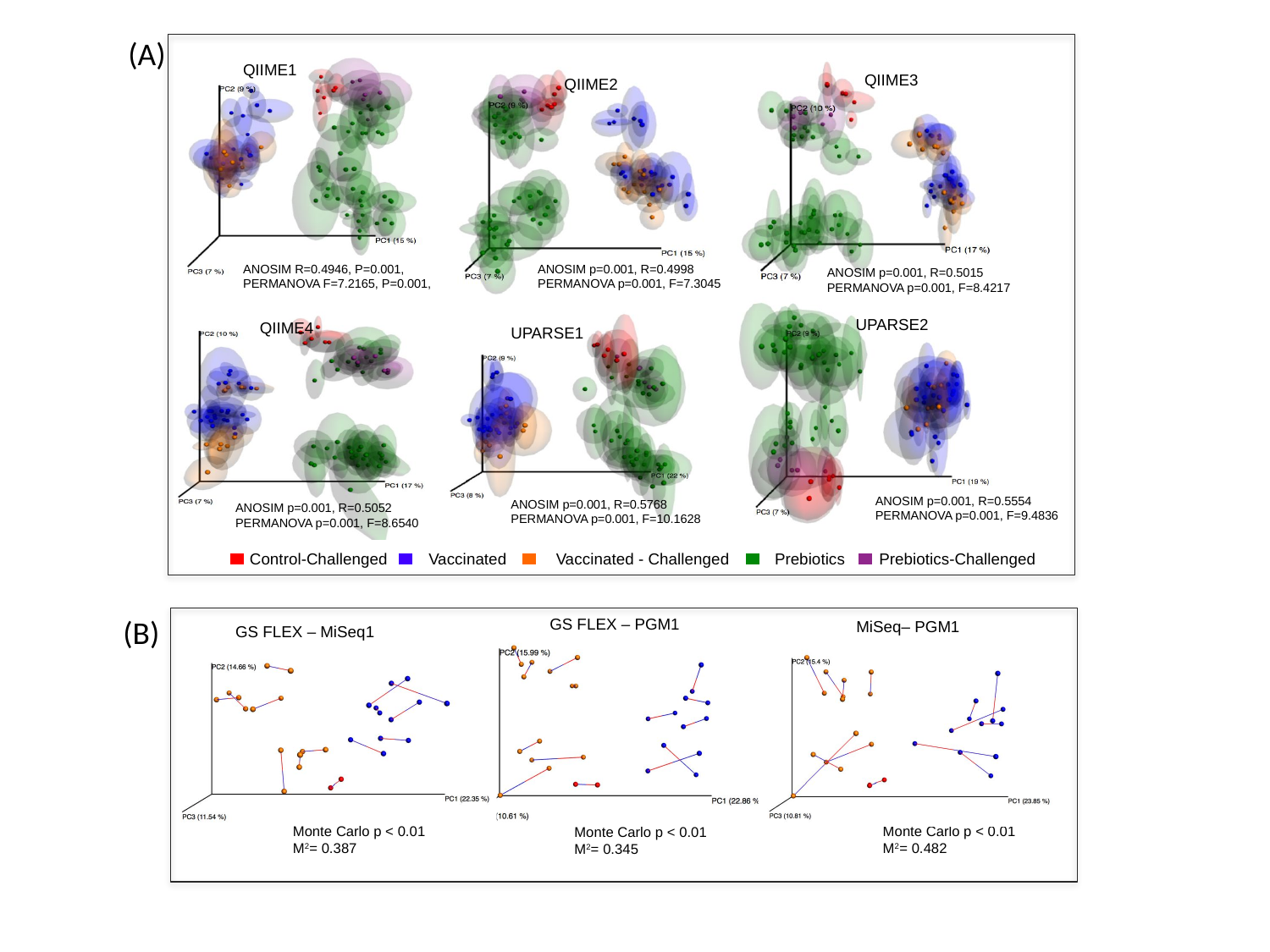

(A)
QIIME1
ANOSIM R=0.4946, P=0.001,
PERMANOVA F=7.2165, P=0.001,
QIIME3
ANOSIM p=0.001, R=0.5015
PERMANOVA p=0.001, F=8.4217
QIIME2
ANOSIM p=0.001, R=0.4998
PERMANOVA p=0.001, F=7.3045
UPARSE2
ANOSIM p=0.001, R=0.5554
PERMANOVA p=0.001, F=9.4836
UPARSE1
ANOSIM p=0.001, R=0.5768
PERMANOVA p=0.001, F=10.1628
QIIME4
ANOSIM p=0.001, R=0.5052
PERMANOVA p=0.001, F=8.6540
Control-Challenged
Vaccinated
Vaccinated - Challenged
Prebiotics
Prebiotics-Challenged
(B)
GS FLEX – PGM1
MiSeq– PGM1
GS FLEX – MiSeq1
Monte Carlo p < 0.01
M2= 0.387
Monte Carlo p < 0.01
M2= 0.482
Monte Carlo p < 0.01
M2= 0.345

## Slide 5
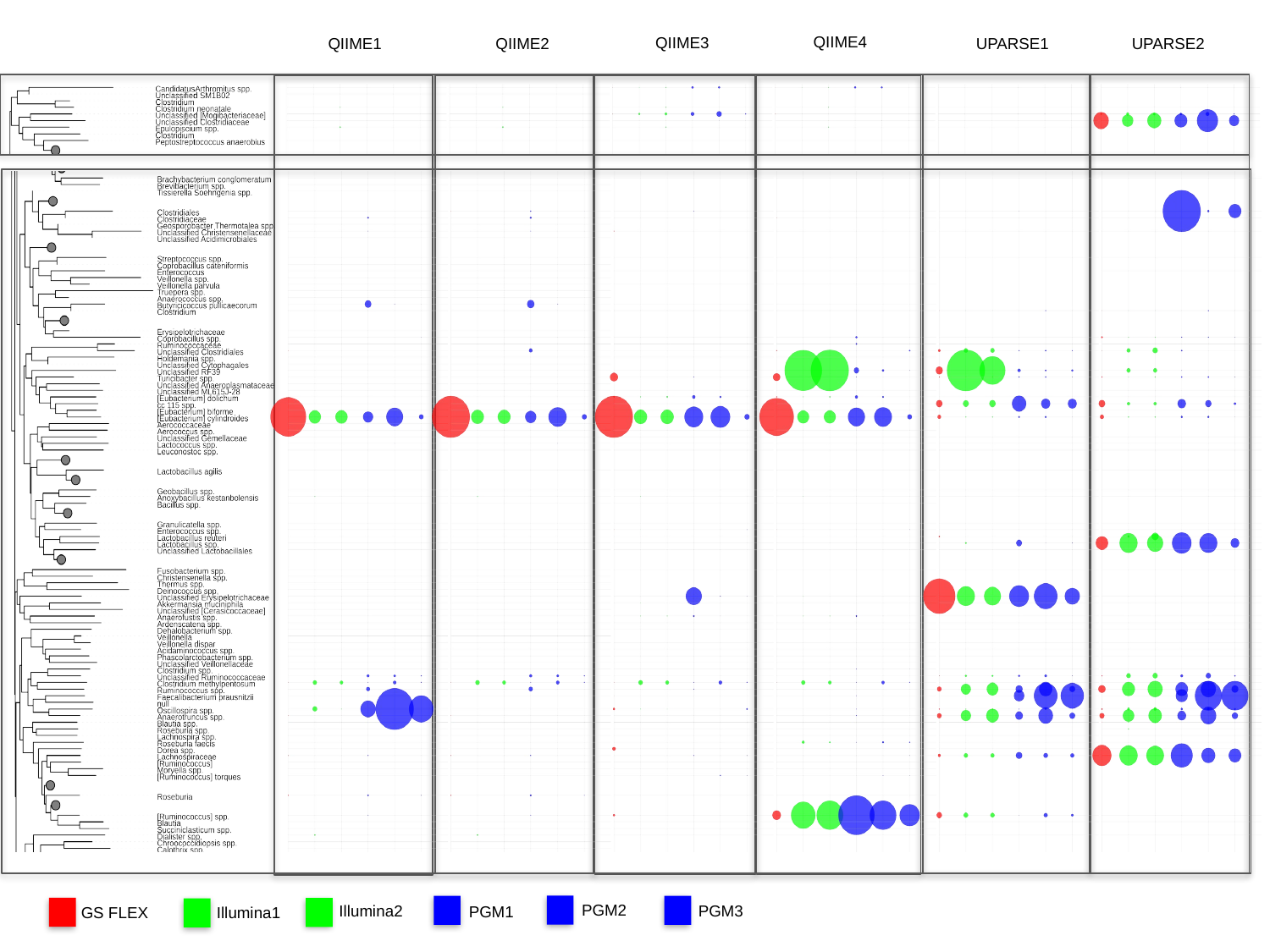

QIIME4
QIIME3
UPARSE1
UPARSE2
QIIME1
QIIME2
PGM2
PGM3
Illumina2
PGM1
Illumina1
GS FLEX

## Slide 6
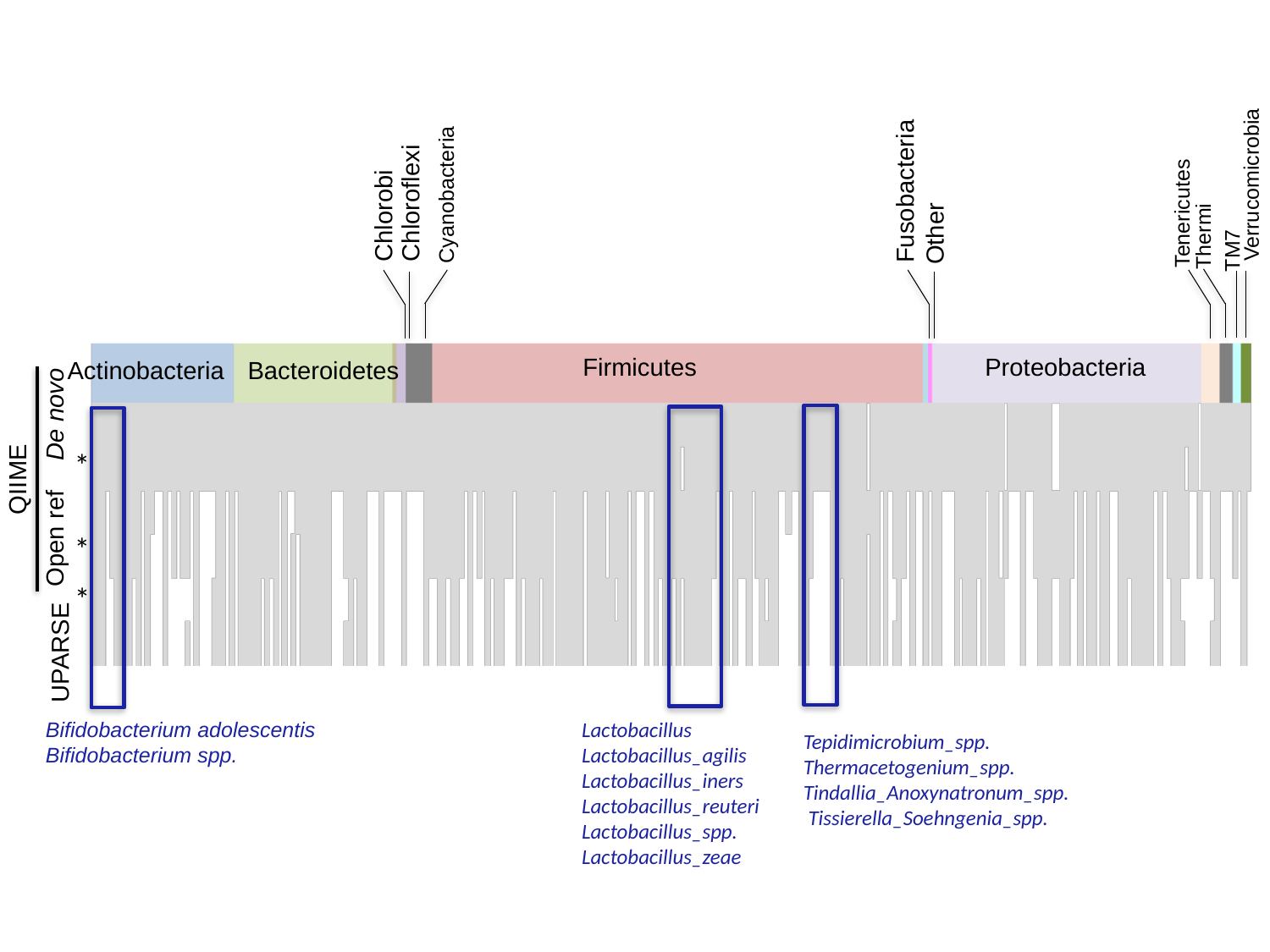

Cyanobacteria
Chloroflexi
Chlorobi
Verrucomicrobia
Fusobacteria
Tenericutes
Other
Thermi
TM7
Firmicutes
Proteobacteria
Actinobacteria
Bacteroidetes
De novo
*
QIIME
Open ref
*
*
UPARSE
Bifidobacterium adolescentis
Bifidobacterium spp.
Lactobacillus
Lactobacillus_agilis
Lactobacillus_iners
Lactobacillus_reuteri
Lactobacillus_spp.
Lactobacillus_zeae
Tepidimicrobium_spp.
Thermacetogenium_spp.
Tindallia_Anoxynatronum_spp.
 Tissierella_Soehngenia_spp.

## Slide 7
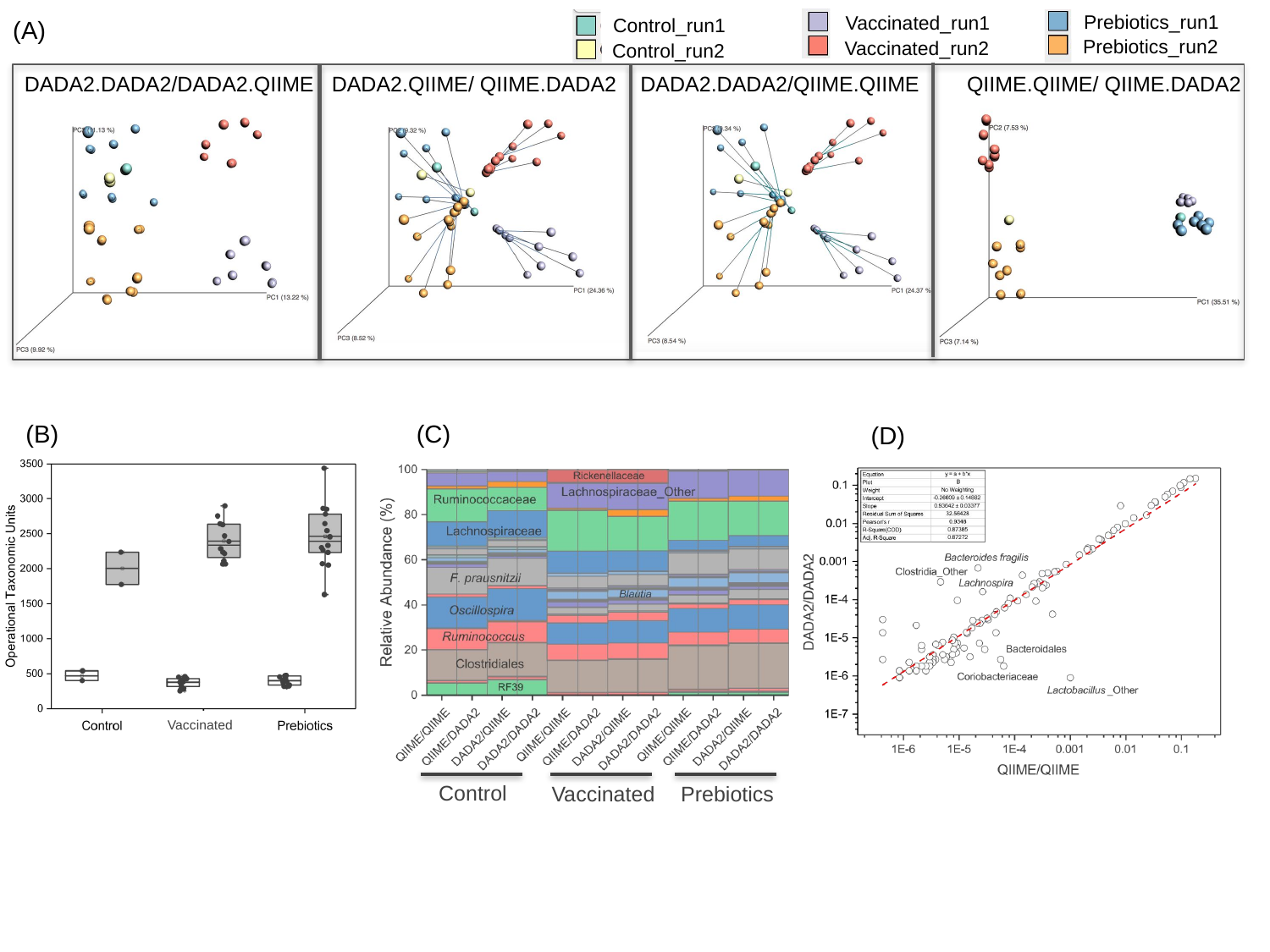

Prebiotics_run1
Vaccinated_run1
Control_run1
Prebiotics_run2
Vaccinated_run2
Control_run2
(A)
DADA2.DADA2/DADA2.QIIME
DADA2.QIIME/ QIIME.DADA2
DADA2.DADA2/QIIME.QIIME
QIIME.QIIME/ QIIME.DADA2
(B)
(C)
(D)
Vaccinated
Control
Vaccinated
Prebiotics
